# Strategies for Building Reference Standards for Autoantibodies

**DOI:** 10.3389/fimmu.2015.00194

**Published:** 2015-04-29

**Authors:** Joanna Sheldon, Alessandra Dellavance

**Affiliations:** ^1^Protein Reference Unit, St George’s Hospital, London, UK; ^2^Research and Development Division, Fleury Medicine and Health Laboratories, São Paulo, Brazil

**Keywords:** reference standards, autoantibodies, quality control, autoimmunity testing, monoclonal antibodies

## Abstract

Producing robust, certified, traceable reference material for autoantibody testing is a vital element in maintaining the validity of results that are generated in the daily clinical laboratory routine. This is a huge challenge because of the high number of variables involved in the detection and measurement of the autoantibodies. The production of such materials is time consuming and needs rigorous attention to detail; this is best achieved by an overarching independent body who will oversee the process in a “not for profit” manner. Much effort has been made to build international standards for quantitative and qualitative assays based on monoclonal antibodies, obtained from affinity purification and plasmapheresis. The big challenge is to respect individual differences in immune response to the same antigen. A promising ongoing initiative is the construction of pools with monospecific samples from different individuals.

## Introduction

The availability of standards or reference materials is central to the generation of reliable and interpretable laboratory which, in turn are vital for accurate patient diagnosis and management. Results from different clinical laboratory measurement procedures should be equivalent within clinically meaningful limits. The characteristics of an ideal reference material are shown in Table 1.

**Table 1:** **Characteristics of a reference material**.

Characteristic	Explanation
**Homogeneous**	Low and stated variability in concentration of the measurand between vials of the material
**Traceable**	Related to a higher-order reference material (usually national or international) through an unbroken chain of comparisons, all with stated uncertainty
**Commutable**	The characteristic of a reference material to behave in a comparable way to the samples (relevant to the intended use of the reference material)
**Available**	There must be sufficient material that is readily available to relevant laboratories or companies over a time period of approximately 5–10 years
**Stable**	The material must be stable over its expected life-span
**Certified**	Ideally, reference material should be certified with stated uncertainties of the various characteristics
**Safe**	Chemically and biologically safe (including tested as negative for HIV and Hepatitis B)
**Ethical**	Where relevant, samples from patients have been collected ethically and with appropriate agreement from the patients

Standardization of invariable analytes that can be prepared as pure substances, e.g., glucose, is straightforward. In contrast, analytes that present subtle variation from one individual to another poise a significant challenge to the building of reference material. This is the case for antibodies, where each individual mounts a polyclonal collection of immunoglobulins that share the property of binding to the antigen of interest. However, they differ regarding the target epitopes, avidity, isotype, etc. This is further complicated by the fact that the multiple available methods for autoantibody detection vary in the ability of detecting different types of immunoglobulins. When the definition of the analyte and the analytical methods are variable, the production of reference material is inadequate (1).

The production of protein standards is more complex because the isolation, purification, and drying steps may all contribute to degradation of the protein making it incomparable with the native protein that would be seen in a clinical sample. Furthermore, almost all peptides and proteins in biological fluids show some degree of molecular heterogeneity and the purification methods sub-fractionate these forms. For simple, single chain proteins, recombinant material may be appropriate although care and attention must be paid to any allotypic variation that may exist but also the minor molecular characteristics, e.g., the glycosylation that may differ between native and recombinant proteins (2). As mentioned above, antibodies or immunoglobulins have a greater degree of molecular heterogeneity than other proteins due to the inherent variability of the antigen binding site, the presence of multiple chains, the presence of immunoglobulin subclasses and variations in affinity and avidity of antibody binding both between and within individuals. An immune stimulus may drive one clone, a few clones or many clones of B cells to produce antibody generating a monoclonal, oligoclonal, or polyclonal response, respectively. Finally, the antigen to which we are trying to measure antibodies is usually a protein with its own variability and molecular heterogeneity. This feature of antibodies is critical to the standardization issue because different methodological platforms vary with respect to the types of immunoglobulins and types of antibody–antigen interactions they are able to detect.

Considering all these issues, alongside the analytical aspects of the detection and quantification of autoantibodies, it is not surprising that standardization of autoimmune serology is a major challenge. It is likely that to have truly robust quantification of autoantibodies, the antigen in question will need to be carefully defined. This may come down even to the molecular domain level as it is reported that antibodies to certain parts of a molecule are associated with less severe disease (3). The analytical platform may also need to be defined as newer methods and technologies are introduced, which adds another source of variation to the analytical process (4). Some methodological platforms favor the presentation of native and conformational epitopes whereas some others preferentially offer denatured linear epitopes. The long-term goal is standardization of clinically relevant antibodies to well characterized autoantigens by thoroughly defined methods but this will be a “step-wise” process based on clinical need and scientific evidence. The most appropriate place to start, however, is with the introduction of reference or standardization material for the antibody.

The majority of clinically relevant autoantibodies are of the IgG class and the starting material for making standards for autoimmune serology will be a biological matrix, e.g., serum (or plasma) from patients known to have antibodies directed against the chosen antigen. Ideally, the autoantibody standards should be obtained from patients with the congnate autoimmune disease; however, the most important factor will be how the (candidate) reference material behaves in comparison to a large panel of samples from patients with and without a stated autoimmune disease or autoantibody. The clonality of autoantibodies is poorly investigated with reports of the presence of monoclonal, polyclonal, and oligoclonal antibodies. Hawa et al. report that in diabetic patients, glutamic acid decarboxylase (GAD) and IA-2 antibodies were usually subclass restricted to IgG1 and were polyclonal although IgM, IgG3, and IgE isotypes were also detected (5). Similarly, Eisenberg et al. report that anti-Sm antibodies in patients with systemic lupus erythematosus are mainly of the IgG1 subclass but with the kappa to lambda light chain distribution similar to that seen in normal polyclonal serum (6).

## The Use of Pooled Sera for Reference Standards for Autoantibodies

Autoantibodies are present in samples from normal patients but usually these are at very low concentration. In contrast, high titer and high affinity autoantibodies are present in patients with certain autoimmune diseases. Samples from such patients are an important source of material to be developed into reference material. The strategy of obtaining large volumes of serum or plasma from a single patient has been successfully used for the establishment of reference material for several autoantibody specificities related to systemic autoimmune rheumatic diseases (7). Plasmapheresis has been used to rapidly remove antibodies from patients and the serum is regarded as the “waste product” that would otherwise be discarded. However, plasmapheresis is less commonly indicated now making this source of samples ethically questionable. The use of single patient material also raises the concern that the autoantibody profile may not be representative of the heterogeneity of patients with that autoimmune disease. Pooling serum samples from a number of patients is likely to generate a material with a wide range of antigenic specificities and likely to be representative of the antibodies that would be seen in many patients. It is worth noting that the production of a certified International Reference Material would be in the order of 2500 vials each containing 1 mL of serum, therefore the starting volume of base material needs to be 3–3.5 L. In order to generate this volume of base material, approximately 150 patients would need to give 50 mL of whole blood, so any initiative to produce autoantibody reference materials pooled from patient samples would need to be a collaboration between different centers or even different countries.

The use of pooled serum for protein standardization is well established; the certified reference preparation ERM-DA470k/IFCC is used across the world for standardization of plasma protein concentrations, e.g., total IgG, IgA, and IgM concentrations (8). However, this material is based on samples from normal donors rather than samples containing abnormal concentrations of analytes. Samples from patients with systemic autoimmune rheumatic diseases often show high concentrations of rheumatoid factor or immune complexes, which may cause interference to varying degrees in immunoassay. Mixing samples from different patients can increase the possibility of interference; therefore, the samples included in the pools should be carefully monitored.

## The Use of Affinity Purified Material for Reference Standards for Autoantibodies

Affinity purification is a powerful technique that can enrich or purify a protein from a complex mixture of proteins. In the context of preparing reference material for autoantibodies, this technique would select the antibodies that bind to the chosen antigen. The antigen (appropriately purified) is chemically immobilized to a support medium, e.g., gel or resin micro-beads packed into a column. The beads are very porous, so proteins can flow freely in and around them providing a huge surface area for interaction between the antigen and the antibodies. The initial binding stage is usually done at physiologic ionic strength and pH then, after a washing stage to remove all unbound proteins, the bound analyte (in this case the autoantibodies) is eluted off the column by dissociating the antibody–antigen binding by changing the pH.

Affinity purification will often remove the potential interfering substances such as rheumatoid factor from the base serum. Material can be made from a number of patient donors and this may be blended to optimize the behavior of the reference material and give long-term consistency. However, there is still a requirement for large volumes of patient serum as the raw material plus significant amounts of the relevant antigen in an appropriate conformational state. The facilities required to produce sufficient affinity purified autoantibodies for a robust “long-term” reference material will be sophisticated and expensive.

There is, however, a very important role of affinity purified autoantibodies; whatever we produce as a potential reference material will need to be value assigned, i.e., to be given a concentration of the analyte in units traceable to a higher-order metrologically based reference measurement system. When we measure autoantibodies, we are actually measuring the amount of IgG that binds onto a defined antigen and ERM-DA470k/IFCC is the certified reference preparation for measurement of IgG in serum. An affinity purified preparation of an autoantibody can be used as a calibrant to assign values of IgG to reference materials produced from pooled serum or serum from other sources. This is the process that has been used for value assignment of the new International Reference Preparation for IgG antibodies to myeloperoxidase, which has now been certified as ERM-DA476/IFCC (9).

## The Use of Monoclonal Cell Cultures for Reference Standards for Autoantibodies

From the homogeneity point of view, monoclonal antibodies present a very attractive base material for the production of standards. The standard producers should have the legal and ethical right to use the cell line for production of material but if the cell line does produce a robust and stable monoclonal antibody, relatively large amounts of the material will be able to be produced at a relatively low cost with long-term consistency. Unlike pooled serum or even plasmapheresis material, monoclonal antibodies should be intrinsically free of interfering substances. However, a monoclonal antibody may not be representative of the spectrum of antibodies seen in patients. Another concern is that the amount of antibody needed may challenge even the most sophisticated cell line production capabilities; if we needed 2500 vials of reference material each containing 1 mL of matrix with an antibody concentration of 50 mg/L, we would need 125 g of the monoclonal autoantibody.

## Ongoing Initiatives

Along with the development of reference material for quantitative assays, some groups are working on improving the standardization of qualitative testing, e.g., indirect immunofluorescence (IIF) for antinuclear antibodies. Mariz et al. showed that the nuclear dense fine speckled (DFS) pattern is the most frequent (45.8%) positive ANA pattern among normal donors when tested using indirect IIF by HEp-2 cells as a substrate (10). The DFS pattern is strongly associated with antibodies to LEDGF/p75 and these are primarily seen as a diagnostically non-specific finding in healthy subjects (10, 11). The recognition of the DFS pattern is an important skill in the ANA testing procedure as correct identification and appropriate reporting should limit misinterpretation by the physician and inappropriate and damaging investigation for the patients. The availability of a quality control or reference material for this pattern, which was readily available, is therefore highly desirable. A 1-L pool of samples from 560 individuals showing high titer (positive at ≥1/640) of the DFS pattern has been collected. This pool has been evaluated to ensure that it preserves the individual heterogeneity of the immune response to the target antigen and represents an opportunity for a proof of concept for the use of pooling samples to construct an autoantibody international standard to be processed on IIF ANA-HEp-2 assay, ELISA, and western blot. This standard shall be available in 2015 by the Autoantibody Standardizing Committee affiliated with the International Union of Immunology Societies (IUIS) at http://asc.dental.ufl.edu/ReferenceSera.html#text.

The process for collecting the anti-DFS samples has been tightly controlled. All samples are coded and kept at −80°C. Individual samples were validated by IIF ANA-HEp-2 assay, anti-LEDGF chemiluminescent assay (BioFLASH, Inova Diagnostics, Inc.). Pentapools (comprising five samples) were sequentially assembled and validated in the BioFLASH assay. Icosapools (comprising four validated pentapools) were sequentially assembled and validated as above. Centumpools (comprising five validated icosapools) were sequentially assembled and validated as above. Finally, five validated centumpools were assembled to constitute the final pool. Pentapools, icosapools, centumpools, and the megapools were also validated in indirect immunofluorescence in HEp-2 cells and in western blot. The semi-quantitative chemiluminescent assay gave us the opportunity to compare the expected and the obtained anti-LEDGF reactivity in all pool combinations. The mean of 425 serum samples was 434.3 (26.1–3080.0) chemiluminescent units (CU) and the mean of 85 pentapools was 484.4 (60.4–25317) CU. Most pools behaved as expected, yielding reactivity quantitatively compatible with the mean of the individual sera, but there were some unexpected deviations in some cases. However, in no case was the deviation significant enough to prevent undisputable anti-LEDGF/p75 reactivity in all methodological platforms.

These results support the development of large pools with multiple individual samples for the establishment of international standards for qualitative tests. However, the extrapolation of this model to other autoantibody specificities must be validated by appropriate studies.

The development of robust standards or certified reference materials is a huge challenge. Success will depend on collaboration between the clinicians, patients, scientists, and the corporate sector with an overarching independent group maintaining the integrity of the process.

## Conflict of Interest Statement

The authors declare that the research was conducted in the absence of any commercial or financial relationships that could be construed as a potential conflict of interest.
